# Opposite Associations between Individual *KIAA0319* Polymorphisms and Developmental Dyslexia Risk across Populations: A Stratified Meta-Analysis by the Study Population

**DOI:** 10.1038/srep30454

**Published:** 2016-07-28

**Authors:** Shanshan Shao, Yanfeng Niu, Xiaohui Zhang, Rui Kong, Jia Wang, Lingfei Liu, Xiu Luo, Jiajia Zhang, Ranran Song

**Affiliations:** 1 Department of Maternal and Child Health and MOE (Ministry of Education) Key Laboratory of Environment and Health, School of Public Health, Tongji Medical College, Huazhong University of Science and Technology, Wuhan, 430030, China; 2 Department of Gastrointestinal Surgery, Union Hospital, Tongji Medical College, Huazhong University of Science and Technology, Wuhan, 430022, China; 3 Department of Epidemiology and Biostatistics, Arnold School of Public Health, University of South Carolina, Columbia, 29208, USA

## Abstract

*KIAA0319* at the DYX2 locus is one of the most extensively studied candidate genes for developmental dyslexia (DD) owing to its important role in neuronal migration. Previous research on associations between *KIAA0319* genetic variations and DD has yielded inconsistent results. It is important to establish a more precise estimate of the DD risk associated with these genetic variations. We carried out a meta-analysis of association studies involving *KIAA0319* polymorphisms and DD risk. The results of pooled analysis indicated that none of the six investigated markers in or near the *KIAA0319* gene are associated with DD. However, a stratified analysis by the study population revealed opposite associations involving *KIAA0319* rs4504469 in European and Asian subgroups. The stratified analysis also showed that the *KIAA0319* rs9461045 minor allele (T allele) has a protective effect in Asians. This meta-analysis has allowed us to establish the effects of specific *KIAA0319* polymorphisms on DD risk with greater precision, as they vary across populations; analyzing one single nucleotide polymorphism at a time could not fully explain the genetic association for DD.

Developmental dyslexia (DD) is one of the most common learning disorders affecting 5–10% of school-aged children worldwide^1^. It is characterized by a specific impairment in reading in the context of adequate intelligence and educational opportunity^1^. Longitudinal evidence indicates that individuals who are diagnosed with DD exhibit long-term, persistent deficits in several cognitive skills and functions, such as phonology, orthography, attention, memory and perception^2^,^3^,^4^,^5^,^6^. These deficits hinder access to knowledge and have an adverse effect on the educational achievement and socioeconomic status of sufferers across the life span^7^,^8^. Although the pathology of DD remains elusive, it is generally considered to be neurobiological in origin^6^,^9^, and is highly heritable^10^,^11^. Initial genetic linkage studies have identified at least nine DD susceptibility loci referred to as DYX1 to DYX9; the most consistently replicated of these is DYX2, which lies at chromosome 6p22.2^12^,^13^,^14^,^15^. This locus has been the subject of numerous studies from which *KIAA0319* has emerged as one of the two strongest candidates (the other is *DCDC2*) for DD^16^,^17^,^18^,^19^,^20^,^21^,^22^.

During the past decade some noteworthy genetic variants in *KIAA0319* have been identified in independent DD samples. An early study by Francks *et al*. identified an underlying quantitative trait locus at chromosome 6p22.2, a 77 kb region spanning the entire *TTRAP* and the first four exons of *KIAA0319,* which was found to be related to DD in two independent UK samples and one US sample; the authors suggested that a three-marker risk haplotype (rs4504469-rs2038137-rs2143340) in this region was associated with DD^16^, thereby identifying *KIAA0319* as a DD-associated gene. Following this study, rs4504469, rs6935076 and their haplotype were observed to be associated with DD in a UK sample^23^. Subsequently, Harold *et al*. not only confirmed that rs4504469 and rs6935076 influenced DD risk, but also identified another promising single nucleotide polymorphism (SNP) candidate for association with DD, rs761100, in a larger UK sample^24^. However, the significant associations of rs4504469 and rs6935076 with DD risk were not confirmed in a US sample studied by Brkanac *et al*.^25^. The positive effect of rs4504469 and rs761100 also did not occur in Toronto samples investigated by Couto *et al*.^19^, in which they found that the alternate allele of rs6935076 was in fact associated with elevated DD risk, which contrasts with findings in the two previously reported UK samples^23^,^24^. The opposite effects were also found for the rs4504469 T allele on DD risk. Three years ago, we combined results from five independent studies of European samples^26^ and showed that the rs4504469 T allele had a protective effect with respect to DD. However, subsequent researches in Asian samples^27^,^28^ showed that the T allele conferred an increased risk of DD.

Nonetheless, *KIAA0319* seems to be an important risk factor for DD, and this conclusion is supported by functional analysis. *KIAA0319* encodes for an integral membrane protein containing a large extracellular domain with four polycystic kidney disease domains, a single transmembrane domain and a small intracellular C-terminus^26^,^29^. Evidence from the cellular level indicates that the KIAA0319 protein may be involved in signaling as well as cell-cell interactions^30^,^31^,^32^. In addition in animal model studies, an embryonic *Kiaa0319* knockdown resulted in disruption to the migration of neocortical neurons leading to the formation of heterotopia in white matter in a subset of animals which also displayed an array of behavioral deficits, including impairments in rapid auditory processing, simple spatial learning^33^ and phoneme processing^34^, similar to those seen in dyslexics. Disrupted neural migration is thought to be an important feature of DD^35^. This evidence suggests that the level of KIAA0319 expression plays an important role in the development of DD.

Several SNPs are involved in regulation of KIAA0319 expression. The risk haplotype rs4504469-rs2038137-rs2143340 identified by Francks *et al*.^16^ has been linked to lower levels of KIAA0319 transcripts^17^,^36^, and rs9461045 has been associated with reduced gene expression^37^ in experimental studies, suggesting that expression of this gene may be affected by the component markers, or other polymorphisms in linkage disequilibrium (LD) with these markers (such as rs3212236 in LD with rs9461045).

The inconsistent results of previous association studies may be due to the limited statistical power of single studies, which means that the modest effects of these variants on DD risk may go undetected owing to the small sample sizes. Meta-analyses are an important tool in the field of association studies, especially when there are lots of published studies and results are inconsistent across studies. We therefore conducted a meta-analysis integrating the results from case-control and transmission/disequilibrium test (TDT) studies in order to derive a more precise estimate of the association between six markers in or near the *KIAA0319* gene (Fig. 1) and DD risk. The criteria for the choice of SNPs were that 1) they had been subject of numerous, inconsistent reports, e.g. rs4504469, rs761100 and rs6935076, or were likely to be related to regulation of KIAA0319 expression, e.g. rs2143340, rs9461045 and rs3212236; and 2) they had been the subject of at least four relevant association studies. We did not include rs2038137 as only three association studies had been published. Because of the inconsistent findings in relation to *KIAA0319* markers and DD risk in different populations we conducted a stratified analysis for all included polymorphisms, with stratification based on the study population.

## Results

### Characteristics of included studies

The literature search and study selection procedures are shown in Supplementary Fig. S1. After comprehensive searching, 40 potentially relevant reports were retrieved; of which, 11 reports met the inclusion criteria. However, two studies reported by Cope *et al*. in ref. 23 and Venkatesh *et al*. in ref. 38 were excluded since the cases largely overlapped with the samples in an analysis by Harold *et al*. in ref. 24 and Venkatesh *et al*. in ref. 27, respectively. Although reports by Elbert *et al*.^39^ and Couto *et al*.^19^ applied the same samples, they focused on different polymorphisms in *KIAA0319*. In addition, the Eicher *et al*.^21^ study’s author kindly replied to our email about asking for original data, and so we also included their data for this meta-analysis^21^. Thus, ten studies were ultimately eligible for this meta-analysis^19^,^21^,^24^,^25^,^27^,^28^,^39^,^40^,^41^,^42^. Because one report^40^ applied two different case groups (dyslexia probands and independent dyslexia) with the same set of controls, we considered it as two case-control studies. Similarly, the Eicher *et al*.^21^ study that contained two DD cohorts was considered as two TDT studies. Table 1 shows the characteristics of the included studies. Finally, there are seven case-control studies with 2,711 cases and 2,991 controls and five TDT studies with 943 families from ten reports included in our meta-analysis.

### Combining the results of case-control and TDT studies

Figure 2 shows the combined results of case-control and TDT studies for six markers in or near the *KIAA0319* gene in association with DD risk. Significant heterogeneity was found for rs4504469, rs761100 and rs6935076 (shown in Table 2); thus, the random-effects models were employed for these three markers and the fixed-effects models were applied for the other markers. In the overall meta-analysis, no statistical evidence of associations was found for any included markers and DD risk, with ORs being equal to 1.04 (95% CI = 0.85–1.27, *P* = 0.706) for rs4504469, 0.95 (95% CI = 0.75–1.21, *P* = 0.689) for rs761100, 1.01 (95% CI = 0.75–1.37, *P* = 0.930) for rs6935076, 0.94 (95% CI = 0.85–1.04, *P* = 0.233) for rs3212236, 0.93 (95% CI = 0.84–1.02, *P* = 0.114) for rs9461045, and 1.03 (95% CI = 0.91–1.16, *P* = 0.653) for rs2143340.

### Stratified analysis

The data were stratified into three subgroups (European, Asian and North American) according to the study population. The results are shown in Table 2. In the stratified analysis, the heterogeneity for rs761100 and rs6935076 remained significant within each subgroup; the heterogeneity for rs4504469 disappeared, and the study population can explain 86.67% of the variance in findings for rs4504469. The stratified analysis showed that rs4504469 T allele had a protective effect with respect to DD in Europeans (OR = 0.90, 95% CI = 0.83–0.99, *P* = 0.028), but in Asians the T allele conferred an increased risk of DD (OR = 1.56, 95% CI = 1.28–1.90, *P* < 0.001). The stratified analysis also revealed that the rs9461045 minor allele (T allele) had a protective effect in the Asian subgroup (OR = 0.82, 95% CI = 0.68–0.98, P = 0.026). The other four markers, rs761100, rs6935076, rs3212236 and rs2143340, were all negatively associated with DD risk in all subgroups.

**Figure 1:**
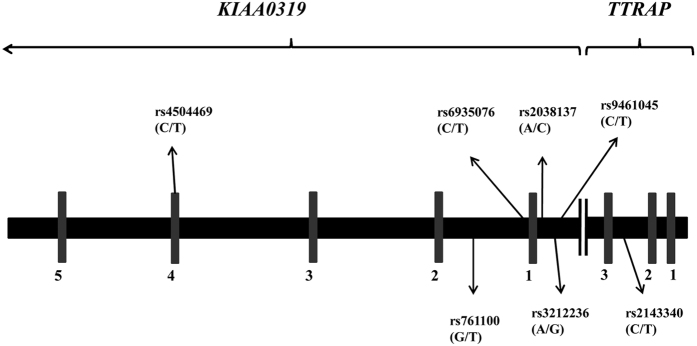
Gene structure and location of six single nucleotide polymorphisms (SNPs) in or near the *KIAA0319* gene in this meta-analysis; one SNP in exon 4 of *KIAA0319*: rs4504469; two SNPs in intron 1of *KIAA0319*: rs6935076 and rs761100; two SNPs located in the promoter region of *KIAA0319* gene: rs9461045 and rs3212236; one SNP in intron 2 of the *TTRAP* gene: rs2143340. *E*xons are represented by *numbered grey rectangles. Black parts* represent gene’s upstream, introns or downstream.

Given that the analysis stratified by the study population explains the opposite direction of effects for rs4504469 but not the rest of the analyzed SNPs, and that this rs4504469 is located further in the 3′ of the gene (as opposed to the others that were clustered around exon1 as shown in Fig. 1), we decided to assess the differences in the LD pattern of different populations for this SNP using snap: https://www.broadinstitute.org/mpg/snap. The results are shown in Fig. 3.

### Sensitivity analyses for combined studies of *KIAA0319* polymorphisms

Given the significant between-study heterogeneity for *KIAA0319* rs4504469, rs761100, and rs6935076 polymorphisms, we conducted a sensitivity meta-analysis to assess the effect of each individual study on the combined ORs for these three markers. Random-effect models were employed since heterogeneity was indicated. The results indicated that whichever studies we omitted the heterogeneity in still existed and the associations were still negative for these three markers. Details are shown in Table 3.

### Publication Bias

Egger’s test was performed to assess the publication bias of the literature. No publication bias was detected for any of the included markers. All the *P* values for Egger’s test were above 0.05 (shown in Table 2).

### Power Analysis

Assuming the odds ratio ranges from 1.15 to 1.35 and the standard deviation is 0.05, the power curve is shown in Supplementary Fig. S2. That means, it can detect the odds ratio greater than or equal to 1.15 at power greater than 0.8. Accordingly, this power curve corresponds to the case with odds ratio 1/1.35 −1/1.15, which means odds ranges 0.74–0.87. That is, it can detect the odds ratio smaller than or equal to 0.87 at power greater than 0.8.

## Discussion

This meta-analysis of associations between six markers in or near the *KIAA0319* gene (rs4504469, rs761100, rs6935076, rs3212236, rs9461045 and rs2143340) and DD risk was based on seven case-control studies comprising a total of 2,711 cases and 2,991 controls and five TDT studies involving 943 families. The results of pooled analysis demonstrated that none of the six markers was associated with DD. A stratified analysis with the study population as the stratification factor revealed that *KIAA0319* rs4504469 had opposite associations with DD risk in European and Asian subgroups. The stratified analysis also revealed that the *KIAA0319* rs9461045 minor allele had a protective effect in the Asian subgroup, but was a risk allele for UK samples^37^. This meta-analysis has allowed us to establish the effects of a specific *KIAA0319* polymorphism on DD risk with greater precision, revealing that they vary with the study population.

Previously published evaluations of the influence of haplotypes in the *KIAA0319* gene on the risk of DD also showed opposite association trends in different samples. Specifically, although under-transmitted haplotypes of rs4504469–rs6935076 were associated with DD in the samples from Cardiff^23^ and Toronto^19^, opposite alleles of these markers were involved in the two samples (2-1 in Cardiff and 1-2 in Toronto- where 1 is major allele and 2 is minor allele for each SNP). In addition, two previous studies that evaluated the influence of the *KIAA0319* gene on reading skills in the general population drew opposite conclusions about the relationship of the 1-1-2 haplotype (rs4504469–rs2038137–rs2143340) to reading performance (positive association in Australia^43^; negative association in England^44^). The two sets of haplotypes overlap in both location and their first component marker, rs4504469 (see Fig. 1).

Lin *et al*. called these opposite findings a ‘flip-flop phenomenon’ and used theoretical modeling to demonstrate that flip-flop patterns of association can occur when the investigated variant is correlated, through interactive effects or linkage disequilibrium, with a causal variant^45^. In other words, examining the association between a single genetic marker and DD risk without taking into account other genetic markers of DD risk and environmental factors correlated with the target susceptibility marker will yield ambiguous results^45^. DD is a complex disease caused by the interaction of various environmental and genetic factors^46^,^47^. The genetic backgrounds of Asian and Western people are different, and they have long lived in different environments. Besides, as Fig. 3 shows, the region in high LD (r^2^ > 0.8) with rs4504469 is not captured by the rest of the analyzed SNPs, and the LD patterns for rs4504469 are different in European and Asian populations. We therefore speculate that there is a functional element in this region and that ethnic differences in inter-marker LD in this region account for the opposite effects of rs4504469, but not the rest of the analyzed SNPs. Of course, differences in inter-marker LD across samples may be due to sampling variation^45^ rather than ethnicity. It is notable that individuals are usually diagnosed with DD if their reading performance falls below a predetermined threshold but there is at present no universally accepted criteria for this threshold and so the existence of subgroups of individuals with DD diagnoses based on different criteria might lead to disparities in the outcomes of association studies^48^.

Neither rs761100 nor rs6935076 were associated with DD risk in the meta-analysis, but these results should be treated with caution because of the repeated presence of heterogeneity suggested by the sensitivity analysis and the stratified analysis. Although we do not know the source of the heterogeneity, it seems arbitrary to simply assume that it is due to false positives. The two SNPs might be correlated, through interactive effects or linkage disequilibrium, with a causal variant, which causing flip-flop associations between these two SNPs and DD risk as discussed above. For example, Ludwig *et al*. found that SNP rs761100 interacted with *DCDC2* haplotype rs793862−rs807701 to affect the quantitative subdimension ‘word reading’^49^ which is the core phenotype for DD in Harold *et al*. study^24^.

**Figure 2:**
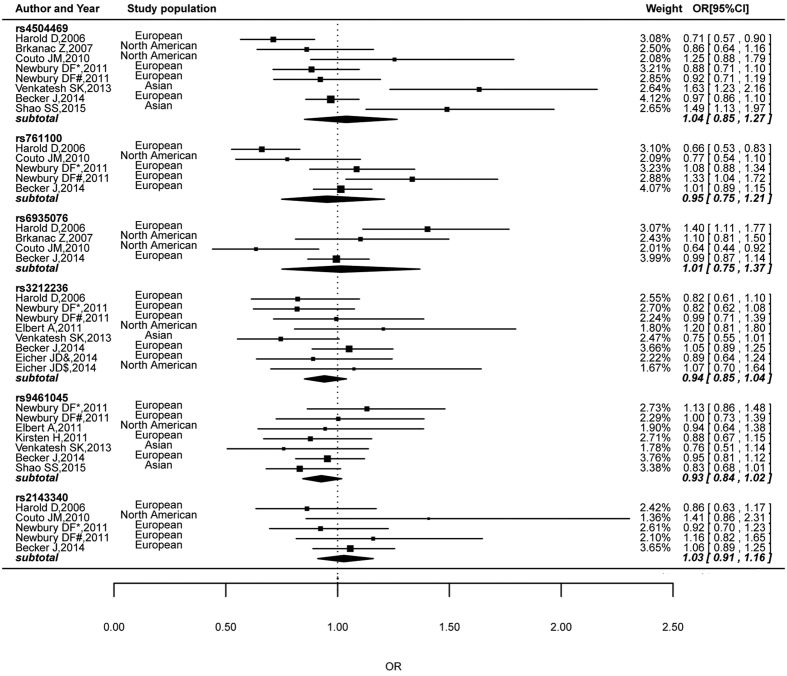
Summary of estimated risk of developmental dyslexia associated with six markers in or near the *KIAA0319* gene based on meta-analysis.

**Figure 3:**
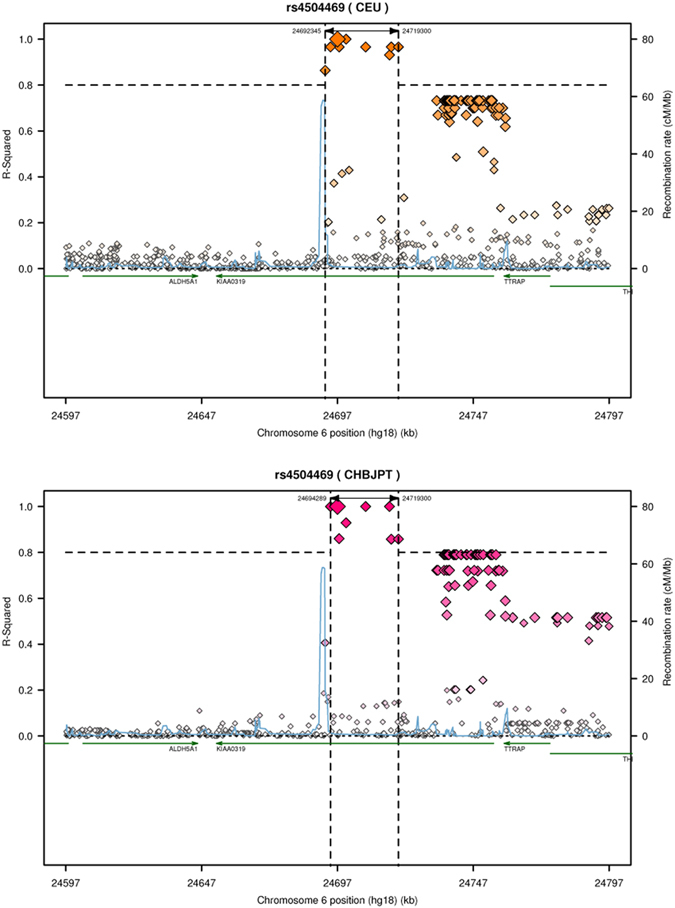
Regional linkage disequilibrium plot for SNP rs4504469 in CEU and JPT + CHB population panels. CEU: Utah residents with Northern and Western European ancestry from the CEPH collection; JPT + CHB: Combined panel of Japanese in Tokyo, Japan and Han Chinese in Beijing, China.

The other three SNPs that were included in this meta-analysis because of their potential effect on *KIAA0319* expression, rs9461045, rs3212236 and rs2143340, were not associated with DD as a discrete trait in each included association studies or in the pooled analysis (see Fig. 2). However, many of the reported associations involving them relate to quantitative reading-related traits^24^,^37^,^43^. Both rs9461045 and rs3212236 are located at the transcription factor-binding site in the promoter region projected by SNP function prediction databases (http://snpinfo.niehs.nih.gov/snpinfo/snpfunc.htm). Rs2143340 is located in the *TTRAP* gene, which encodes a tumor necrosis factor receptor-associated protein which inhibits nuclear factor-kappa B (NF-κB) transcription^50^. NF-κB transcription is important for long-term potentiation and synaptic plasticity which are associated with learning and memory^23^. Based on this we suggest that these three SNPs might be implicated in specific reading skills rather than general reading disability. If possible, a systematic review of research on the effect of *KIAA0319* markers on quantitative reading-related traits should be priority for future research.

In summary, the meta-analysis clarified the associations between *KIAA0319* polymorphisms and DD risk and suggested that the associations between single SNPs and DD risk vary according to the study population. There may be multiple genetic variants (rather than a single SNP) that contribute to DD risk. Further primary research and updated meta-analysis of findings on *KIAA0319* is needed to explore the combined effect of multiple genetic polymorphisms and environmental factors on DD risk. In complex diseases the effect of single causal variant is very weak, and the sample sizes in our analysis were relatively small, especially in the stratified analysis. More studies with larger samples in a particular ethnic group are needed to confirm our findings and identify true causal variants via fine-mapping.

## Methods

### Search strategy and identification of relevant studies

We searched PubMed, EMBASE, and ISI Web of Science databases for published articles up to May 2015 using the keywords “KIAA0319”, “dyslexia or reading disability”, “polymorphism or variant” without language restrictions. References of the retrieved articles were also scanned. Reviews, comments, and letters were also checked for additional studies.

The following inclusion criteria had to be fulfilled: (1) either case-control or TDT study design; (2) *KIAA0319* polymorphisms and DD risk; (3) allele frequencies on *KIAA0319* polymorphisms in case and control groups for case–control studies, and numbers of transmitted alleles from heterozygous parents to affected offspring for family-based studies. For the study that only meet the first two conditions, we emailed its corresponding author to asking for the original data mentioned in the third condition. Animal studies, reviews, simple commentaries and meetings were excluded. Study overlap was eliminated by selecting the report with a complete design or larger sample size.

### Data extraction

All data were extracted in dependently by two reviewers (S. Shao & X. Zhang). The following information was extracted from the eligible studies: first author’s name, year of publication, study population, study design, and *KIAA0319* polymorphisms. The counts of alleles in case and control groups in case-control studies and the number of transmitted alleles from heterozygous parents to affected offspring in family-based studies were extracted or calculated in the included studies.

### Statistical analysis

All statistical analyses were conducted using the metafor package (version 1.9-5) in R (version 3.2.3; http://www.r-project.org/). Briefly, data from each case-control and TDT study were extracted and sorted as per the metafor package instructions. Odds ratios (ORs), 95% confidence intervals (95%CIs) and standard errors (SEs) were calculated for individual study based on allele data. The between-study heterogeneity was assessed by a *χ*^*2*^-based Cochran’s Q-statistic test (the heterogeneity was considered significant at *P* < 0.10). A fixed-effects model was used to apply data from studies when heterogeneity was negligible; otherwise, a random-effects model was applied. For the synthesis of case-control and TDT studies, Kazeem and Farrall^51^ outlined a methodological improvement for achieving integration by a fixed-effects approach, and then Nicodemus subsequently extended this method to the random-effects model^52^. Stratified analyses were conducted according to the study population. Publication bias was assessed by Egger’s test^53^. In addition, a sensitivity analysis was performed to evaluate the influence of each study on the overall estimate. The power is calculated based on power formula of the mixed model for individual regression coefficients^54^. All *P* values were two-tailed with a significance level at 0.05, except for Cochran’s Q-statistic test.

## Additional Information

**How to cite this article**: Shao, S. *et al*. Opposite Associations between Individual *KIAA0319* Polymorphisms and Developmental Dyslexia Risk across Populations: A Stratified Meta-Analysis by the Study Population. *Sci. Rep.* **6**, 30454; doi: 10.1038/srep30454 (2016).

## Supplementary Material

Supplementary Information

## Figures and Tables

**Table 1:** Characteristics of included studies.

Study	Publication year	Study population	Design type	Case/control (family)	KIAA0319 marker
Harold D	2006	Cardiff of UK	Case/control	350/273	rs761100, rs4504469, rs3212236, rs6935076, rs2143340
Brkanac Z	2007	Washington	TDT	191	rs4504469, rs6935076
Couto JM	2010	Toronto	TDT	156	rs4504469, rs6935076, rs761100, rs2143340
Newbury DF^#^	2011	UK	Case/control	188/363	rs9461045, rs761100, rs4504469, rs3212236, rs2143340
Newbury DF^*^	2011	UK	Case/control	331/363	rs9461045, rs761100, rs4504469, rs3212236, rs2143340
Elbert A	2011	Toronto	TDT	156	rs9461045, rs3212236
Kirsten H	2011	German	case–control	272/548	rs9461045
Venkatesh SK	2013	Indian	Case/control	210/256	rs9461045, rs4504469, rs3212236
Becker J	2014	Eight European countries	Case/control	958/1150	rs9461045, rs761100, rs4504469, rs3212236, rs6935076, rs2143340
Eicher JD^$^	2014	Colorado	TDT	292	rs3212236
Eicher JD^&^	2014	Italy	TDT	304	rs3212236
Shao SS	2015	Chinese	Case/control	402/401	rs9461045, rs4504469

^*^Case group is composed of independent dyslexia cases;

^#^Case group is composed of dyslexia probands;

^$^ and ^&^ represent two independent cohorts from Colorado and Italy, respectively.

**Table 2:** Meta-analysis for associations between KIAA0319 polymorphisms and DD.

KIAA0319 markers	No.^*^	Study population	MAF in controls^#^	Sample sizecases/controls (families)	OR (95% CI)	*P* value	Heterogeneity		Model for meta-analysis	Egger’s test	
							Q statistic (*P* value)	I^2^ (%)		z	*P* value
rs4504469	8	Total	0.13~0.43	2439/2443 and 347	1.04 (0.85–1.27)	0.706	32.375 (<0.001)	82.27	**Random**	1.034	0.301
	4	European	0.40~0.43	1827/1786	**0.90** (**0.83–0.99)**	**0.028**	5.427 (0.143)	45.11	**Fix**	−1.552	0.121
	2	Asian	0.13, 0.26	612/657	**1.56** (**1.28–1.90)**	**<0.001**	0.210 (0.647)	0.00	**Fix**	–	–
	2	North American	–	347	1.03 (0.71–1.48)	0.889	2.535 (0.111)	60.55	**Fix**	–	–
rs761100	5	Total	0.39~0.46	1827/1786 and 156	0.95 (0.75–1.21)	0.689	20.002 (<0.001)	83.28	Random	−0.472	0.637
	4	European	0.39~0.46	1827/1786	0.99 (0.75–1.31)	0.951	18.207 (<0.001)	87.18	Random	0.089	0.929
	1	North American	–	156	0.78 (0.55–1.10)	0.155	–	–	–	–	–
rs6935076	4	Total	0.35, 0.36	1308/1423 and 347	1.01 (0.75–1.37)	0.930	13.957 (0.003)	83.65	Random	−0.794	0.427
	2	European	0.35, 0.36	1308/1423	1.17 (0.83–1.63)	0.371	6.273 (0.012)	84.06	Random	–	–
	2	North American	–	347	0.85 (0.49–1.45)	0.540	5.135 (0.023)	80.53	Random	–	–
rs3212236	8	Total	0.17~0.35	2037/2042 and 752	0.94 (0.85–1.04)	0.233	7.812 (0.349)	17.94	Fix	−0.433	0.665
	5	European	0.17~0.19	1827/1786 and 304	0.95 (0.84–1.06)	0.327	3.675 (0.452)	15.81	Fix	−1.324	0.185
	1	Asian	0.35	210/256	0.75 (0.55–1.01)	0.056	–	–	–	–	–
	2	North American	–	448	1.14 (0.85–1.53)	0.375	0.150 (0.698)	0.00	Fix	–	–
rs9461045	7	Total	0.18~0.42	2361/2718 and 156	0.93 (0.84–1.02)	0.114	4.727 (0.579)	0.00	Fix	−0.094	0.925
	4	European	0.18~0.19	1749/2061	0.98 (0.87–1.10)	0.678	1.841 (0.606)	0.00	Fix	0.368	0.713
	2	Asian	0.39, 0.42	612/657	**0.82** (**0.68**–**0.98)**	**0.026**	0.154 (0.695)	0.00	Fix	–	–
	1	North American	–	156	0.94 (0.64–1.39)	0.770	–	–	–	–	–
rs2143340	5	Total	0.15~0.16	1827/1786 and 156	1.03 (0.91–1.16)	0.653	3.898 (0.420)	0.01	Fix	0.489	0.625
	4	European	0.15~0.16	1827/1786	1.01 (0.89–1.14)	0.903	2.243 (0.523)	0.00	Fix	−0.479	0.632
	1	North American	–	156	1.41 (0.86–2.31)	0.175	–	–	–	–	–

^*^The number of studies.

^#^The range of minor allele frequency (MAF) in the control group of the included studies.

**Table 3:** Sensitivity analysis of pooled ORs for *KIAA0319* polymorphisms and DD.

Study omitted	OR(95% CI)	*P*	*P* for heterogeneity	I^2^
For rs4504469				
Harold D, 2006	1.10 (0.91–1.33)	0.347	0.001	77.85
Brkanac Z, 2007	1.07 (0.86–1.33)	0.570	0.000	84.56
Couto JM, 2010	1.02 (0.82–1.26)	0.882	0.000	84.73
Newbury DF^*^, 2011	1.07 (0.85–1.34)	0.576	0.000	83.63
Newbury DF^#^, 2011	1.06 (0.84–1.33)	0.624	0.000	84.75
Venkatesh SK, 2013	0.97 (0.82–1.16)	0.754	0.004	74.21
Becker J, 2014	1.05 (0.83–1.33)	0.664	0.000	81.25
Shao SS, 2015	0.99 (0.81–1.20)	0.897	0.001	79.73
For rs761100				
Harold D, 2006	1.05 (0.89–1.24)	0.531	0.082	55.63
Couto JM, 2010	0.99 (0.75–1.31)	0.951	0.000	87.18
Newbury DF^*^, 2011	0.92 (0.68–1.24)	0.583	0.000	86.18
Newbury DF^#^, 2011	0.88 (0.70–1.11)	0.286	0.004	78.69
Becker J, 2014	0.93 (0.68–1.28)	0.668	0.000	83.87
For rs6935076				
Harold D, 2006	0.91 (0.68–1.22)	0.524	0.051	72.22
Brkanac Z, 2007	0.98 (0.64–1.50)	0.922	0.001	90.26
Couto JM, 2010	1.14 (0.92–1.42)	0.232	0.043	65.97
Becker J, 2014	1.01 (0.64–1.59)	0.962	0.002	85.56

^*^Case group is composed of independent dyslexia cases.

^#^Case group is composed of dyslexia probands.
