# Supplementary material for: Opposite Associations between Individual KIAA0319 Polymorphisms and Developmental Dyslexia Risk across Populations: A Stratified Meta-Analysis by the Study Population
Source: Sci Rep. 2016 Jul 28;6:30454. doi: 10.1038/srep30454 (PMC4964335; doi:10.1038/srep30454)
Supplement: Supplementary Information [file srep30454-s1.pdf]

Opposite Associations between Individual *KIAA0319* Polymorphisms and Developmental Dyslexia Risk across Populations: A Stratified Meta-Analysis by the Study Population

Shanshan Shao[1#], Yanfeng Niu[2#], Xiaohui Zhang[1], Rui Kong[1], Jia Wang[1], Lingfei Liu[1], Xiu Luo[1], Jiajia Zhang[3], Ranran Song[1*]

[#] S. S. and Y.N. contributed equally to the work.

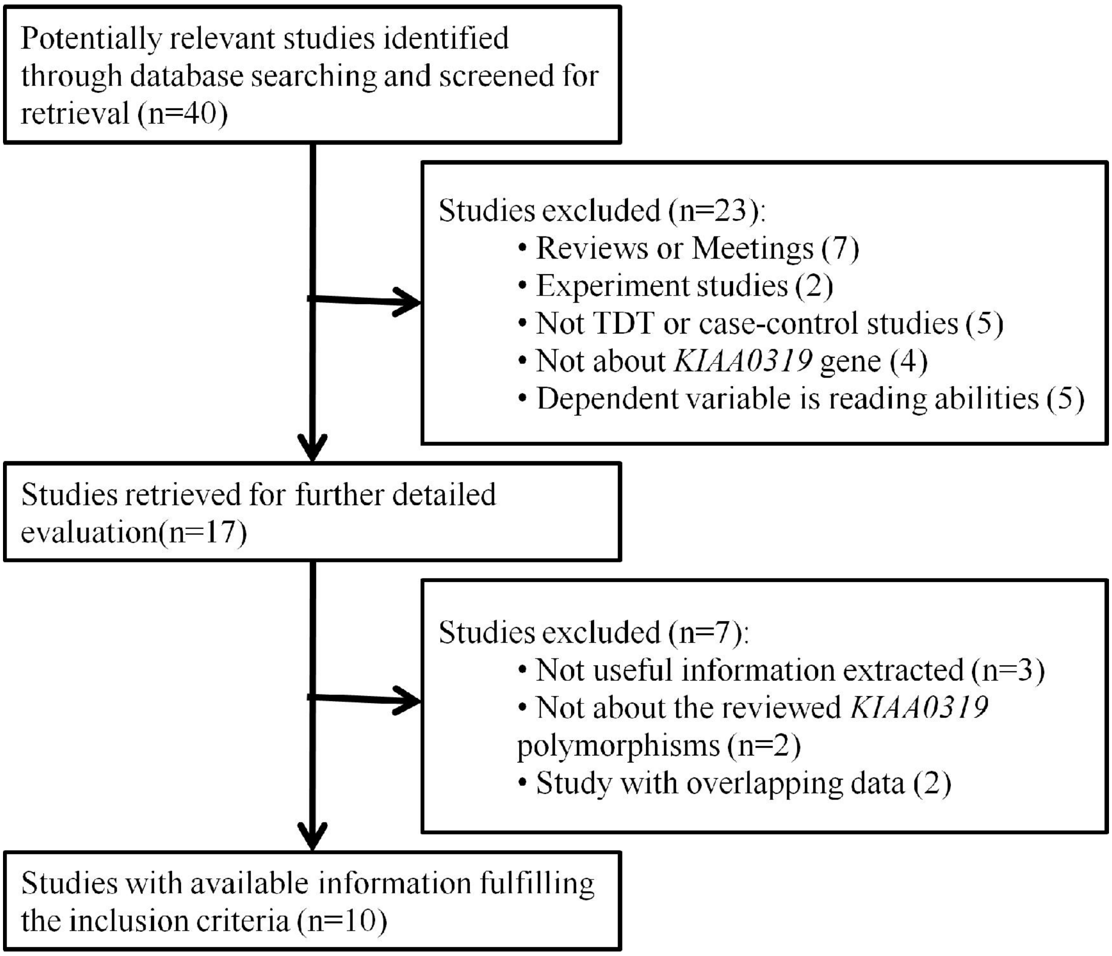

Supplementary Figure S1 The comprehensive literature search and study selection procedures for *KIAA0319* polymorphisms and developmental dyslexia risk

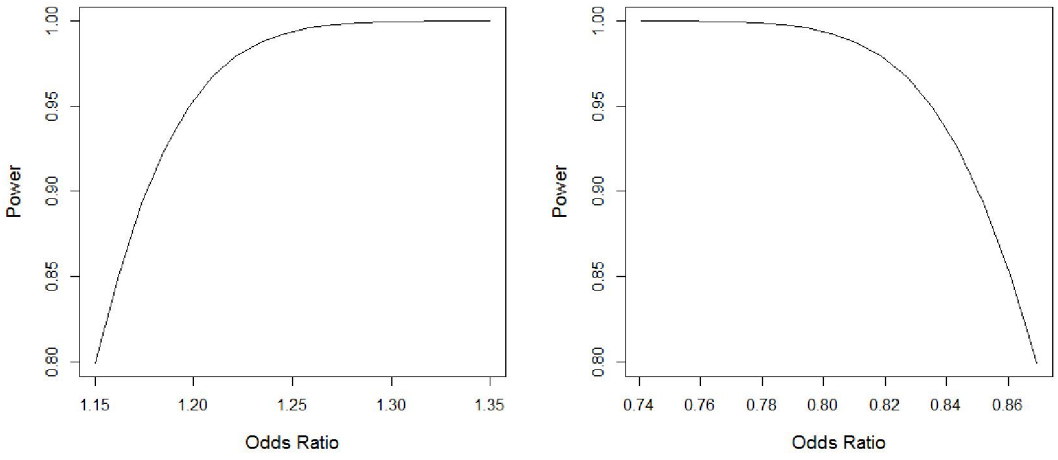

Supplementary Fig. S2 The power curves given the standard deviation of 0.05 and the odds ratio from 1.15 to 1.35 (left) or from 0.74 to 0.87 (right)
